# Elderly patients undergoing laparoscopic radical cystectomy benefit from dynamic D-dimer measurement in combination with vascular ultrasonography: reduction of VTE risk and improvement of clinical outcomes

**DOI:** 10.3389/fmed.2025.1623198

**Published:** 2025-07-07

**Authors:** Wenbo Gao, Haihong Ye, Yangkai Xu

**Affiliations:** Department of Urology, Ningbo Urology and Nephrology Hospital, Ningbo City, Zhejiang, China

**Keywords:** bladder cancer, cystectomy, thromboembolism, D-dimer, screening

## Abstract

**Background:**

Venous thromboembolism (VTE) is serious in elderly patients undergoing laparoscopic radical cystectomy and bilateral pelvic lymph node dissection. We compared the results of two VTE prophylaxis protocols: dynamic D-dimer measurement in combination with ultrasonography screening in Experimental Group (EG), and conventional VTE prophylaxis in historical control group (HCG).

**Methods:**

Between January 2022 and January 2024, the elderly patients undergoing such surgeries in EG received dynamic measurement of plasma D-dimer at admission and at 1, 3, 7, and 14 days after surgery in combination with ultrasonography screening, and commensurate VTE mechanical prophylaxis measures. Between January 2019 and December 2021, elderly patients in HCG underwent conventional prophylaxis and mechanical measures. And they were observed carefully for VTE symptoms, with Doppler ultrasonography being performed only in patients with clinical suspicion for VTE. The incidence of VTE event, major postoperative complications, major bleeding rate, and evaluation of activities of daily living within 30 days postoperatively were compared.

**Results:**

The preoperative and intraoperative parameters were similar between the two groups. In EG, dynamic D-dimer measurements revealed a distinct temporal declining pattern. In HCG, VTE was detected in five patients out of 98 patients (5/98, 4.08%); and in EG, eight patients were found to have DVT (8/109, 7.34%; *p* = 0.04). The incidence of symptomatic VTE was significantly lower in EG than in HCG (one and five cases, respectively, 0.9% vs. 5.1%, *p* = 0.04), and the incidence of postoperative asymptomatic VTE was higher in the EG than in the HCG (seven and 0 cases, respectively, 6.4% vs. 0%). The incidence of major complications was similar between the two groups (*p* = 0.61), with similar result regarding the incidence of major bleeding (*p* = 0.55). The average Barthel index score in EG was 81.0 points, significantly higher than 78.3 points of the HCG (*p* = 0.03), and the result demonstrated a faster recovery of activities of daily living in the Experimental Group.

**Conclusion:**

Our results demonstrated that postoperative dynamic D-dimer and ultrasonography measurement in elderly patients can better monitor the risk of VTE, identify asymptomatic thrombosis at an early stage, optimize the timing of intervention and improve clinical outcomes, without resulting in more complications or major bleeding. Elderly patients undergoing laparoscopic radical cystectomy could benefit from such strategies.

## 1 Introduction

Bladder cancer (BC) remains a prevalent malignancy among the elderly population, with radical cystectomy being the cornerstone treatment for localized muscle-invasive disease (MIBC). However, postoperative complications, particularly venous thromboembolism (VTE), pose significant threats to the patients' recovery and survival. Previous studies indicated that up to 50% of malignancy patients exhibit hypercoagulability, with VTE incidence further amplified by surgical stress and advanced age (1). Older age has been related to worse outcomes in VTE, probably because aging diminishes physiological reserve, as well as plus more comorbidities, thus necessitates proactive preventive strategies (2).

Contrast venography is the gold standard for diagnosis of VTE. However, the technical issues, expense, invasiveness and use of potentially hazardous contrast agents restrict its actual application (3). In particular, it is not suitable for routine screening of asymptomatic VTE patients. D-dimer, a fibrin degradation product, has emerged as a critical biomarker reflecting coagulation- fibrinolysis imbalance. Clinical studies have shown that D-dimer measurement for predicting VTE usually has a high sensitivity of 80%−100%, but a low specificity of approximately 20%−60% (4); and it could be elevated by a number of different conditions, such as cancer, infection, trauma and heart failure, etc. (5). Despite its prognostic value, the static D-dimer assessments (including the traditional VTE prevention strategy- Caprini score stratification) may ignore the dynamic bleeding/coagulation risk changes (such as inflammatory factors, prolonged bed rest) during the perioperative period. In addition, D-Dimer levels increase with age, and its levels in elderly patients are often higher than the conventional critical value (500 μg/L). For elderly patients, sole use of this assay is likely to be uninformative (6). More importantly, in elderly patients undergoing major surgery, non-thrombotic elevations of D-dimer are frequently observed due to surgical trauma and age-related endothelial dysfunction. This diagnostic ambiguity creates a critical clinical dilemma: over-treatment with anticoagulants based solely on D-dimer levels may expose frail patients to hemorrhagic risks, while delayed intervention could lead to catastrophic thromboembolic events.

To address this challenge, emerging evidence suggests integrating morphological assessment through vascular ultrasonography. And “Chinese guidelines for the prevention and treatment of thrombotic diseases” also recommends to use high-sensitive D-dimer detection to screen patients; if the result is positive, further ultrasonography examination should be performed (7). Doppler ultrasonography offers distinct advantages in this context: ① it enables direct visualization of venous thrombi, particularly in lower extremities where most postoperative VTE originates; ② the dynamic assessment of venous compressibility and flow patterns provides real-time hemodynamic information complementary to biochemical markers; ③ its non-invasive nature permits repeated examinations. It has been found that monitoring of D-dimer levels in combination with vascular ultrasonography could enhance early VTE detection and intervention in several surgical fields, for example, spinal cord injury, gynecologic surgery (8) and emergency patients (9, 10). Therefore, it is assumed that the biomarker-imaging synergy may be especially pertinent in laparoscopic radical cystectomy (LRC) patients, as the operation's unique risk profile predisposes to iliac vein compression syndromes often undetectable by single D-dimer test. Currently, research on the integrating application of dynamic D-dimer test and vascular ultrasonography for screening of VTE in LRC patients, especially elderly patients, is lacking.

The present study aims to evaluate the efficacy of a dynamic monitoring protocol- combining serial D-dimer measurements and vascular ultrasonography- in the detection of VTE among elderly patients undergoing LRC, as compared with historical cohorts. By addressing the gap in real-time coagulation monitoring, we endeavored to find whether this vulnerable population could benefit from such strategies.

## 2 Materials and methods

### 2.1 Study design

This clinical study compared an experimental group with historical control group. Patients undergoing LRC for bladder cancer from January 2022 to December 2024 were enrolled as the Experimental Group (EG, *n* = 119), with the intervention and venous thromboembolism (VTE) prophylaxis protocols described as follows. A historical control group (HCG, *n* = 106) was formed by patients receiving LRC between January 2019 and December 2021 under conventional VTE prophylaxis protocols. Baseline characteristics, including preoperative- age, sex, comorbidities; intraoperative- operative duration and estimated blood loss; and postoperative: dynamic D-dimer measurements and ultrasonography findings were recorded.

The inclusion criteria were: age ≥60 years, pathologically confirmed bladder malignancy. Patients were excluded if they received neoadjuvant therapy, serum creatinine≥2 mg/dl, or with a diagnosis of VTE within 6 months or already anticoagulated preoperatively, or with concurrent malignancies, or BMI ≥ 40 kg/m^2^. LRC surgeries were performed by two skilled surgeons (both of them had experience of more than 50 cystectomy operations), according to a standardized procedure. Pelvic lymphadenectomy- including obturator field nodes, internal and external iliac nodes up to the common iliac artery- was performed in all patients.

**Figure 1:**
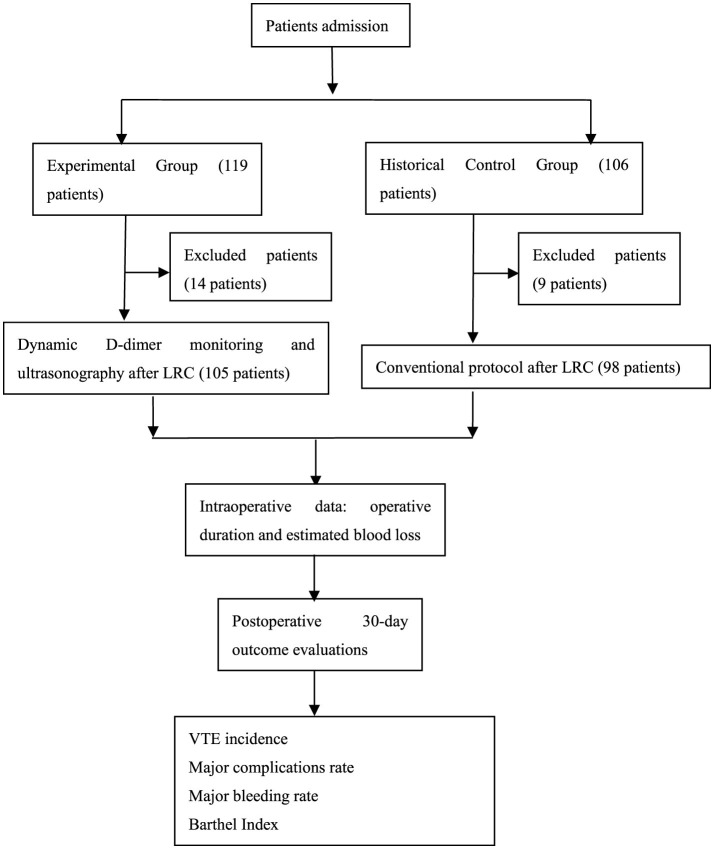
Study flow chart.

This study- retrospective and based on already recorded data- was conducted in accordance with the principles of the Declaration of Helsinki, and the study protocol was approved by the ethics committee of our hospital. Informed consent was obtained from all patients. The study flow chart is seen in Figure 1.

### 2.2 Intervention protocols

#### 2.2.1 Experimental group

All patients received measurement of plasma D-dimer concentrations at admission and at 1, 3, 7, and 14 days after surgery. The detection of D-dimer was performed by use of the immunoturbidimetry assay (CA-7000; Sysmex, Kobe, Japan). Postoperatively, if D-dimer levels increased consecutively or exceeded the age-adjusted D-dimer cutoff value: age (years) × 10 ng/ml fibrinogen equivalent units (FEU), bilateral lower extremity compression ultrasonography was performed. The ultrasonography measurements were performed by two skilled Ultrasound doctors, and both of them had experiences of more than 500 cases of ultrasound examination. If D-dimer levels remained below the age-adjusted D-dimer cutoff value, bilateral lower extremity compression ultrasonography was scheduled on postoperative day 10.

**Figure 2:**
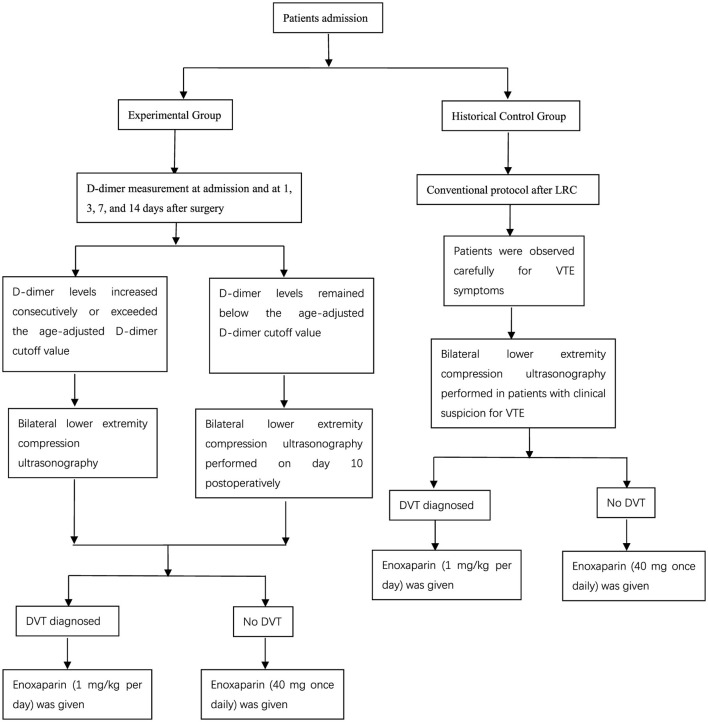
Intervention protocols.

DVT was diagnosed when Doppler ultrasonography showed loss of compressibility of the vein, absence of venous flow and existence of intraluminal echogenicity. If a patient had symptoms suspective of PE, such as dyspnea, tachypnoea or chest pain, then pulmonary CT angiography was performed to confirm or exclude PE. For patients with DVT or PE, therapeutic-dose low-molecular-weight heparin (LMWH)- enoxaparin 1 mg/kg per day- was initiated immediately. Other patients received routine prophylactic-dose anticoagulation therapy: enoxaparin 40 mg once daily (seen in Figure 2).

As a basic prophylaxis, all patients received graduated compression stockings and intermittent pneumatic compression devices starting 8 h postoperatively. If VTE was confirmed, the compression stockings and pneumatic compression devices were removed.

#### 2.2.2 Historical control group

Between January 2019 and December 2021, our elderly LRC patients received conventional VTE prophylaxis protocols: they were observed carefully for VTE symptoms, for example, calf swelling, pain, skin-color change, and calf cramps (for DVT); chest pain, dyspnea, tachypnea, tachycardia, and decrease in blood pressure (for PE). Doppler ultrasonography was performed only in patients with clinical suspicion for VTE. As a routine measure, prophylactic-dose LMWH (enoxaparin 40 mg once daily) was initiated 2–3 days postoperatively. Additionally, identical mechanical prophylaxis measures were performed as mentioned above. And any other institutional changes occurred during this period (e.g., surgeon experience, anesthesia protocols, enhanced recovery pathways).

### 2.3 Outcome measures

The primary outcome was the incidence of VTE event (symptomatic and asymptomatic; including DVT and PE) within 30 days postoperatively.

The secondary outcomes included major postoperative complications, major bleeding rate, and evaluation of activities of daily living (ADL), assessed as of 30 days postoperatively. Given the inherent limitations of retrospective data collection in capturing minor postoperative events, this study specifically focused on major postoperative complications- Clavien–Dindo (CD) grade III: requiring surgical/endoscopic/radiologic intervention, or higher events. Major bleeding was defined according to the definition provided by the International Society on Thrombosis and Haemostasis (ISTH): bleeding with symptomatic presentation and fatal bleeding and/or bleeding in a critical area or organ (e.g., intracranial, intraspinal, intra-ocular, retroperitoneal, intra-articular or pericardial bleeds, or intramuscular bleeds with compartment syndrome) and/or bleeding causing a fall in hemoglobin levels by at least 2 g/dl (1.24 mmol/L) leading to transfusion of two or more units of whole blood or red cells (11). Evaluation of ADL was conducted by use of the Barthel index (BI) questionnaire (Table 1) (12), which is used for assessing the independence of geriatric patients in self-care (feeding, bathing, dressing, toileting) and mobility (transfers, stairs) across 10 domains of activities. Each domain has two to four response categories, with total scores ranging from 0 (fully dependent) to 100 (fully independent). The BI survey was performed in person or by phone. Two medical assistants performed the BI assessments and they were blinded to the clinical study.

**Table 1:** Barthel index questionnaire.

**Feeding**	**Dressing**
0 = unable	0 = dependent
5 = needs help cutting, spreading butter, etc.	5 = needs help, but can do about half unaided
10 = independent (food provided within reach)	10 = independent (including buttons, zips, laces, etc.)
**Bladder**	**Transfer**
0 = incontinent or catheterized and unable to manage	0 = unable–no sitting balance
5 = occasional accident (max once per 24 h)	5 = major help (one or two people, physical), can sit
10 = continent (for over 7 days)	10 = minor help (verbal or physical)
	15 = independent
**Bathing**	**Bowel**
0 = dependent	0 = incontinent (or needs to be given enemata)
5 = independent (or in the shower)	5 = occasional accident (once/week)
Toilette use	10 = continent
0 = dependent	
5 = needs some help but can do something alone	
10 = independent (on and off, dressing, wiping)	
**Grooming**	**Stairs**
0 = needs help with personal care	0 = unable
5 = independent face/hair/teeth/shaving (implements provided)	5 = needs help (verbal, physical, carrying aid)
	10 = independent up and down
**Mobility**	**Total scoring system**
0 = immobile	0–20: “total dependency”
5 = wheelchair independent, including corner, etc.	25–60: “severe dependency”
10 = walks with help of one person (verbal or physical)	65–90: “moderate dependency”
15 = independent (but may use any aid, e.g., stick)	95: “slight dependency”
	100: “total independency”

### 2.4 Statistical analysis

Descriptive statistics were used to examine baseline characteristics in both groups. The quantitative data were presented as mean ± standard deviation. The independent samples Student's *t*-test and the chi-squared test or Fischer exact test (sample < 5) were used to evaluate the differences between the groups for quantitative and qualitative variables, respectively. All statistical analyses were performed using SPSS software version 18.0, with a *p*-value of < 0.05 being considered statistically significant.

## 3 Results

### 3.1 Baseline results

At the beginning, this study involved 119 patients in the EG and 106 patients in the HCG. However, 14 and eight patients were, respectively excluded from the EG and HCG, due to incomplete or missing data, or already on anticoagulation therapy for mechanical heart valve, previous history of DVT, etc. Ultimately, 105 patients were included in the EG and 98 patients were included in the HCG. The preoperative and intraoperative parameters are listed in Table 2, including age, sex, Charlson comorbidity index, operative duration, estimated blood loss, preoperative D-dimer levels as well as a comparison of these variables between the two groups.

**Table 2:** The preoperative and intraoperative parameters of the two groups.

**Variables**	**Experimental group**	**Historical control group**	***p*-Value**
*n*	109	98	
Age (years)	69.0 ± 5.9	68.3 ± 4.9	0.39
**Gender**			
Male, *n* (%)	82 (75.2%)	76 (77.6%)	0.69
Female, *n* (%)	27 (24.8%)	22 (22.4%)	0.30
**Charlson comorbidity index**			
0	48 (44.0%)	42 (42.9%)	0.31
1–2	41 (37.6%)	38 (38.8%)	
≥3	20 (18.4%)	18 (18.3%)	
Operative duration (min)	225.8 ± 28.7	233.2 ± 27.4	0.06
Estimated blood loss (ml)	123.1 ± 32.9	116.1 ± 27.2	0.10
Preoperative D-dimer (ng/ml)	178.4 ± 43.6	186.8 ± 45.8	0.17

The pathologic results are listed in Table 3, which demonstrated that in both groups, most patients were graded as T2 and staged as N0. No significant difference was seen between the two groups with respect to tumor grade and stage. Moreover, in both groups, all LRC procedures were performed laparoscopically without open conversion.

**Table 3:** The pathologic results of both groups.

**Variables**	**Experimental group**	**historical control group**	***p-*Value**
*n*	109	98	—
**T grade**			
T1 (%)	19 (17.4%)	12 (12.2%)	0.31
T2 (%)	75 (68.8%)	67 (68.4%)	
T3 (%)	15 (13.8%)	19 (19.4%)	
**N stage**			
N0 (%)	85 (78.0%)	82 (83.7%)	0.29
N1 (%)	20 (18.3%)	11 (11.2%)	
N2 (%)	4 (3.7%)	5 (5.1%)	

### 3.2 Dynamic change of D-dimer levels and the patients of above the age-adjusted cut-off value in the experimental group

**Figure 3:**
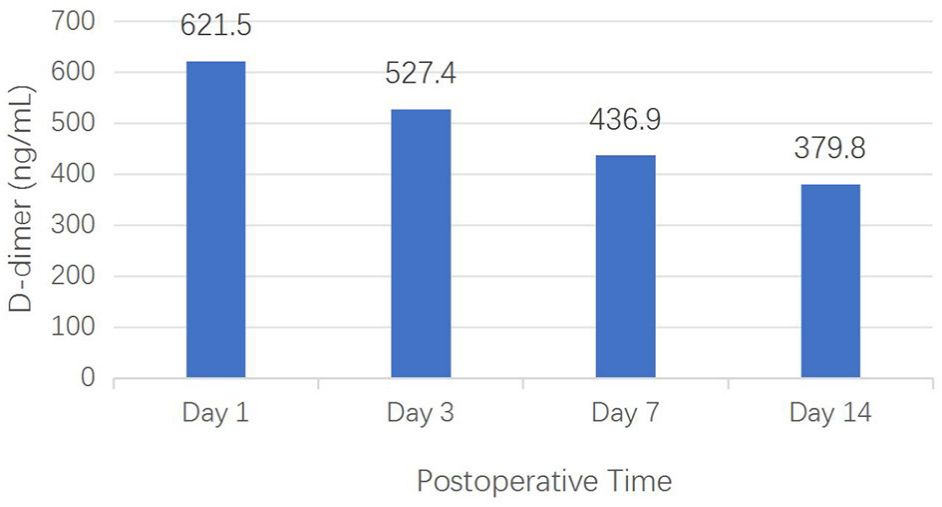
Dynamics of D-dimer levels in the experimental group.

As demonstrated in Figure 3, serial D-dimer measurements revealed a distinct temporal pattern in EG: a gradual declining trend from 621.5 ng/ml at postoperative day 1 (with a standard deviation of 54.7) to 379.8 ng/ml at postoperative day 14 (a standard deviation of 70.1). Note: because the D-dimer levels in the HCG were not regularly measured, its temporal changes could not be evaluated.

When stratified by age groups, the cohort comprised 67 (61.5%) patients aged 60–69 years, 32 (29.4%) aged 70–79 years, and 10 (9.1%) aged 80–89 years. As can be seen from the Table 4, the number of patients undergoing LRC decreases with age. Furthermore, according to the age-adjusted D-dimer cut-off values, a total of 26 patients exhibited elevated levels exceeding their age-specific thresholds. And the percentage of the patients with elevated levels in its respective age range group is also shown in Table 4.

**Table 4:** The number of patients stratified by age group and those of above the age-adjusted cut-off value in the experimental group.

**Variables**	**Age range (years)**		
	**60–69**	**70–79**	**80–89**
Number of patients (%)	67 (61.5%)	32 (29.4%)	10 (9.1%)
Number of patients above the age-adjusted cut-off value (%)	13 (19.4%)	9 (28.1%)	4 (40%)

### 3.3 Incidence of postoperative VTE

**Figure 4:**
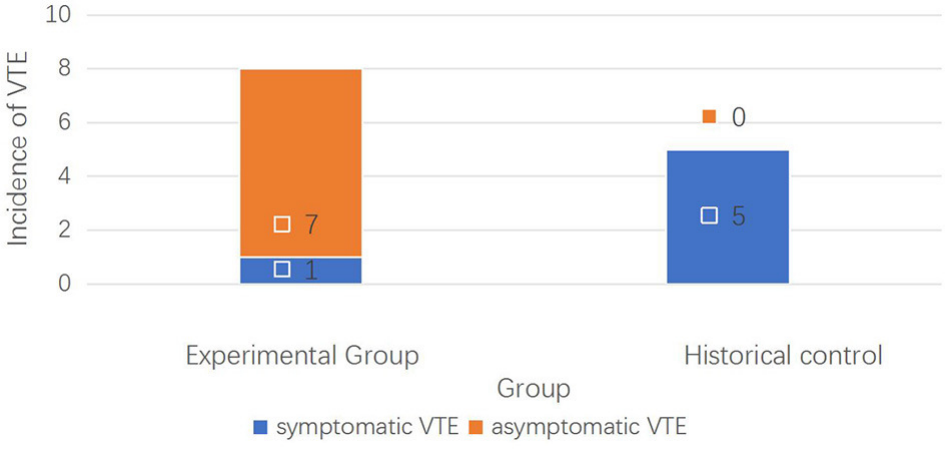
Incidence of symptomatic and asymptomatic VTE events in both groups.

Out of the 98 patients in HCG, VTE was detected in five patients (5/98, 4.08%), with four cases of DVT and one case of PE. While in EG, eight patients were found to have DVT (8/109, 7.34%), among whom one experienced postoperative symptomatic DVT and seven had asymptomatic VTE, without PE. The results showed the incidence of symptomatic VTE was significantly lower in the EG than in HCG (one and five cases, respectively, 0.9 vs. 5.1%, *p* = 0.04), as shown in Table 5. However, the incidence of postoperative asymptomatic VTE was higher in the EG than in the HCG (seven and 0 cases, respectively, 6.4% vs. 0, *p* = 0.04), which suggested that the early detection rate of asymptomatic VTE was higher by use of our dynamic monitoring protocol (Figure 4). Subsequently, the occurrence of symptomatic VTE was greatly reduced, and the risk of PE decreased accordingly. After appropriate treatment, these patients recovered well.

**Table 5:** Comparison of postoperative VTE between the two groups.

**Variables**	**Experimental group**	**Historical control group**	***p*-Value**
VTE, *n*, %	8 (7.6%)	5 (5.1%)	0.04
Symptomatic VTE, *n*, %	1 (0.9%)	5 (5.1%)	0.04

### 3.4 Incidence of major postoperative complications and major bleeding

Table 6 presents a summary of the 30-day postoperative complications graded by CD and major bleeding in both groups. In total, three patients (2.8%) in EG and three patients (3.1%) in HCG experienced major complications (CD grade ≥III) requiring another intervention under general anesthesia. There was no difference between the two groups (*p* = 0.61), suggesting that the adverse events were not increased although blood tests were performed more than before.

**Table 6:** Comparison of 30-day postoperative major complications between the two groups.

**Variables**	**Experimental group**	**Historical control group**	***p*-Value**
Clavien–Dindo ≥ III grade, *n*, %	3 (2.8%)	3 (3.1%)	0.61
Major bleeding complication, *n*, %	3 (2.8%)	2 (2.0%)	0.55

With respect to postoperative major bleeding, one patient underwent re-operation about an hour after the operation in the EG. Careful re-examination revealed that a small vessel in the pelvic cavity was not ligated completely. And other two patients received two units of red blood cells transfusion. The incidence of postoperative major bleeding was 2.8%. Whereas in HCG, 1 patient experienced transfusion of three units of red blood cells and 1 patient was re-admitted to hospital due to a large ecchymoma. Therefore, the incidence of postoperative major bleeding was 2.0%. There was no significant difference in the incidence of major bleeding between the two groups (*p* = 0.55), suggesting that although dynamic adjustment of anti-coagulation intensity in EG, the incidence of major bleeding was not elevated. No patient died with 30 days postoperatively in both groups.

### 3.5 Evaluation of ADL: Barthel index score

The average preoperative BI scores of both groups were 89.8 and 91.1 points for EG and HCG, respectively (Table 7); which indicated that most patients had good self-care ability before surgery. Thirty days postoperatively, for patients in EG, the average BI score was 81.0 points, which was significantly higher than that of the HCG (78.3 points; *p* = 0.03). The result demonstrated a faster recovery of ADL in EG. This might be related to early postoperative mobility which resulted from early intervention to reduce symptomatic VTE events (for example, swelling or pain of the lower limbs, or restrictions from anticoagulation therapy) in EG. Conversely, for HCG patients, the reduced BI score probably meant that VTE delayed functional recovery.

**Table 7:** Comparison of Barthel index scores between the two groups.

**Barthel index**	**Experimental group**	**Historical control group**	***p*-Value**
Preoperative	89.8 ± 5.1	91.1 ± 5.6	0.09
30 days postoperatively	81.0 ± 7.9	78.3 ± 8.8	0.03

## 4 Discussion

Radical cystectomy in elderly patients might confer a greater risk of VTE compared with other major surgical interventions due to several characteristics of aging population: ① blood vessel walls become more fragile; ② endothelial functions decline; ③ blood viscosity increases; ④ levels of baseline coagulation activity elevates (13) or anticoagulant mechanisms weaken, such as reduced antithrombin III; ⑤ reduced physical activity results in insufficient daily activities, leading to slow venous blood flow and increased thrombosis risk; and ⑥ decreased muscle mass (sarcopenia) could affect venous return (14). All these factors contribute to elderly patients' high risk for VTE, especially for those with greater surgical trauma and longer postoperative immobilization. In urology, LRC involves pelvic procedures, fixed body posture during the long operation time and high pressure CO_2_ insufflations of the abdomen, further increasing the risk of VTE. Compared to the general population, elderly BC patients are especially susceptible to thrombosis development owing to many factors, including inflammatory response, hypercoagulable state, long-term indwelling catheters, as well as the postoperative tendency to limit mobilization predisposing to venous stasis (15). Numerous studies showed that patients with malignant tumors have a four to seven times higher risk of VTE (16). Furthermore, older age could be related to worse outcomes of VTE, which is also partly associated with an increasing comorbidity burden and a decreased cardiopulmonary reserve; and the VTE treatment complexity with bleeding risk is greatly increased under conventional anticoagulant therapy. Consequently, earlier and more reliable diagnoses of VTE (DVT and PTE) are necessary for the affected elderly patients. Screening methods for VTE mainly comprise risk prediction tools, imaging tests and D-dimer test (11). Of these, D-dimer has been proven useful for evaluating the risk of VTE in patients with oncologic surgery (17). Nevertheless, D-dimer levels increase with age. In elderly patients, its level is often higher than the conventional cut-off value (500 ng/ml), precluding the specificity of D-Dimer test. Researchers have conducted numerous clinical studies to reduce its false positive rate. One meta-analysis including 12,497 patients compared the conventional D-dimer cutoff values (< 500 ng/ml) with age-adjusted values- age (year) × 10 ng/ml- for patients aged >50 years. The result demonstrated the specificity of the conventional cut-off value declined with increasing age, from 57.6% for patients aged 51–60 years to 39.4, 24.5, and 14.7% in patients aged 61–70, 71–80, and >80 years, respectively. Whereas the age-adjusted cut-off values showed higher specificity−62.3, 49.5, 44.2, and 35.2%, respectively; and the sensitivities of the age-adjusted cut-offs remained >97% in older patients (5). In our study, we adopted the age-adjusted value of D-dimer test. Preoperatively, the D-dimer levels were similar in both groups. Postoperatively, in EG, serial age-adjusted values showed highly suggestive: when the levels increased consecutively or were consistently higher, it suggested high risk for VTE and an urgent need for Doppler ultrasonography. Our results demonstrated that dynamic D-dimer test could be used to predict thrombosis at an early stage, making up for the lack of intermittent ultrasonography screening. Meanwhile, it showed that the age-adjusted cut-off value of D-dimer is an effective threshold to trigger further imaging examination, and this was in agreement with the study of Ortega et al. (2). Wu et al. also revealed that the age-adjusted cut-off values of D-dimer considerably elevated its specificity without altering sensitivity in patients aged >50 years with suspected PTE (3).

Owing to differences in the demographic data and surgical approaches, the reported postoperative VTE incidence varied from 0.54 to 8% after radical cystectomy (RC) (15). In one study that examined patients who underwent urologic tumor surgery, An et al. found that patients undergoing radical cystectomy were most likely to suffer VTE, with an incidence of 4.3%, which was similar with our data in the HCG (4.08%). But in our EG, the result showed that by use of dynamic D-dimer measurement and ultrasonography, through accurately identifying high-risk periods, seven cases of asymptomatic VTE were identified. Its advantage lies in the fact that the disease can be detected and treated in the early stage to prevent further damage or serious complications. This conclusion is consistent with that of Kumagai et al. (18) who also showed that ultrasonography combined with D-dimer tests identified more asymptomatic DVT events than D-dimer alone in patients with acute spinal cord injury. Moreover, the American College of Chest Physicians evidence-based clinical practice guidelines on the diagnosis of DVT also recommends that D-dimer assay should not be used as a sole test to rule out DVT in aging patients (7).

In Imamura et al.'s clinical study (19), they evaluated whether a preoperative D-dimer value could identify patients who would benefit from lower limb venous ultrasound (LLVU). In the clinical indication group, LLVU was performed only when DVT was clinically suspected, and they detected DVT in only 0.7% of patients. While in the D-dimer-orientated group, LLVU was performed in the patients with an abnormal preoperative D-dimer value, and they detected DVT in 2.3% of patients. And they found that postoperative symptomatic VTE was significantly decreased at both 3 and 6 months after surgery (*p* = 0.041 and 0.020, respectively). Our results were similar with theirs, when asymptomatic DVT was early detected and treated. Actually, the invisibility of asymptomatic thrombus is potential dangerous to patients. Undiagnosed asymptomatic VTE (e.g., DVT) may progress to symptomatic thrombosis or lead to pulmonary embolism (PE) if a clot dislodges. Particularly, PE is probably the most dangerous complication of VTE and can cause sudden death; and untreated asymptomatic VTE may result in thrombus extension or chronic sequelae (e.g., postthrombotic syndrome), impairing quality of life. The main benefit of detecting asymptomatic VTE lies in preventing avoidable complications and death, especially for high-risk patients. Furthermore, early detection allows timely interventions, significantly reducing the risk fatal complications, ischemic stroke and myocardial infarction, as well as preventing potential mortality. In our study, a high proportion of DVT (6.4%) were asymptomatic in EG, and no PE occurred in this group. Conversely, one PE occurred in HCG. This result suggested that the asymptomatic VTE events were treated as early as possible, resulting in decreased incidence of symptomatic VTE events. We believe that early and precise diagnosis can shift the treatment strategy from postevent intervention to preventive intervention, without increasing adverse reactions or major bleeding. Literature has shown that early intervention could significantly improve patient function retention rates: the incidence of postthrombotic syndrome (PTS) decreases from 30 to 8%; ② the 5-year survival rate for chronic thromboembolic pulmonary hypertension (CTEPH) increases from 30 to 85%; and ③ the incidence of cognitive dysfunction in patients with cerebral microthrombi is reduced by 65% (20). With the deep integration of omics technology and artificial intelligence, VTE prevention and control would achieve comprehensive optimization from molecular mechanisms to macro-management. On the other hand, conducting these measurements might increase the economic cost, but compared to the medical burden associated with thrombosis, this additional cost is likely to be affordable. And this can be studied in further clinical research.

In our study, we investigated the influence of VTE on patients' health status by use of the BI score which has gained popularity in assessment of ADL abilities. The BI was developed by Dorothea Barthel with the aims to monitor advances in self-care and mobility skills, and measure levels of patient independence. It consists of 10 domains, and is currently recommended for detecting clinically relevant changes of elderly patients. The change of BI score in elderly surgical patients may reflect functional recovery and complication risk (21). Furthermore, it can indirectly reflect the venous blood flow dynamics of geriatrics by assessing their daily living ability (such as eating, transferring, walking, and so on). Existing studies are mainly focusing on the application of BI in nursing and caring (12). In our study, we observed that preoperative BI score was similar between the two groups. After operation, the BI in HCG was 78.3 points, which was significantly lower than that of EG, indicating that the postoperative activity abilities were improved with lower occurrence of symptomatic VTE events. This can be attributed to the fact that functional limitations caused by VTE were correlated with immobility, which is a key component of Virchow's triad (stasis, hypercoagulability, endothelial injury) (22). Our data also implied that understanding the influence of immobilization after VTE on the functioning of the elderly patients is of great importance; as such patients are rather vulnerable, especially after major surgical attack. In addition, researchers also found that psychological burden (such as “potential thrombosis” anxiety) could be alleviated by health education and providing personalized risk communication based on early screening of thrombosis (23), although we didn't conduct relevant clinical study in this regard. In addition, we included patients ≥60 years as elderly patients. We know this is not the common definition of “old patient” for Western countries. But in China, according to “the Law of the People's Republic of China on the Protection of The Rights and Interests of the Elderly,” in Article 2, it clearly stipulates that the term “elderly” refers to citizens over the age of 60.

### 4.1 Limitations

There are still several limitations in this study. Firstly, it is a single-centered and retrospective study, with small sample size. Retrospective studies rely on pre-existing data, which may involve non-randomized patient selection, thus selection bias could be rendered, or uncontrolled confounding factors could make it difficult to isolate causal relationships. And the use of a historical control group (HCG) might exacerbate selection or information bias, as perioperative management practices may have changed over time. But in our study, we didn't change other factors except the prophylaxis protocols. The results require validation in larger patient cohorts and in prospective studies. Secondly, the follow-up period was short-term, limiting to the first 30 postoperative days. It is probably difficult to ascertain whether this protocol is useful enough for patients undergoing LRC to avoid postoperative symptomatic VTE in the long run. Thirdly, part of the tests in Section 3.2 dynamic changes of D-dimer levels and the number of patients stratified by age group and those of above the age-adjusted cut-off value- were performed only in the experimental group. Because these tests were irregularly or not performed in HCG, corresponding comparisons could not be made between the two groups. And the higher incidence of asymptomatic VTE in the experimental group is expected by design and does not demonstrate improved clinical outcomes unless linked to meaningful endpoints (e.g., fewer PE, mortality, rehospitalization). Additionally, the adverse effects produced by enoxaparin must be noticed; however, we did not investigate the complications of enoxaparin in our study.

## 5 Conclusions

Elderly patients with BC are at increased risk for VTE, especially when undergoing LRC. Thromboprophylaxis remains challenging in such patients. Our results demonstrated that postoperative dynamic D-dimer and ultrasonography measurement can better monitor the risk of VTE, identify asymptomatic thrombosis at an early stage, optimize the timing of intervention and improve clinical outcomes, without resulting in more complications or major bleeding. The present study suggests that dynamic D-dimer assay in combination with ultrasonography is of great importance in clinical practice, especially for aging patients receiving LRC. The cost-effectiveness of this procedure in a wider population needs to be further validated.

## Data Availability

The original contributions presented in the study are included in the article/supplementary material, further inquiries can be directed to the corresponding author.
